# Home-based exercise and physical activity intervention after kidney transplantation: impact of exercise intensity (PHOENIX-Kidney). Protocol for a multicentre randomized controlled trial

**DOI:** 10.1093/ckj/sfaf114

**Published:** 2025-04-24

**Authors:** Stefan De Smet, Marieke Vandecruys, Jasmine De Beir, Sofie Leunis, Karsten Vanden Wyngaert, Marie Renier, Henriette de Loor, Kaatje Goetschalckx, Maarten Naesens, Delphine De Smedt, Francis Verbeke, Ingeborg Stalmans, Steffen Fieuws, Griet Glorieux, Jan Van Eijgen, Hanne Van Criekinge, Jeroen Raes, Sabina De Geest, Wim Van Biesen, Dirk Kuypers, Evi Nagler, Patrick Calders, Diethard Monbaliu, Véronique Cornelissen, Amaryllis H Van Craenenbroeck

**Affiliations:** Department of Movement Sciences, Exercise Physiology Research Group, KU Leuven, Leuven, Belgium; Department of Rehabilitation Sciences, Group Rehabilitation for Internal Disorders, KU Leuven, Leuven, Belgium; Department of Microbiology, Immunology and Transplantation, Nephrology and Renal Transplantation Research Group, KU Leuven, Leuven, Belgium; Department of Microbiology, Immunology and Transplantation, Abdominal Transplantation, KU Leuven, Leuven, Belgium; Department of Microbiology, Immunology and Transplantation, Nephrology and Renal Transplantation Research Group, KU Leuven, Leuven, Belgium; Department of Rehabilitation sciences, Ghent University, Ghent, Belgium; Department of Microbiology, Immunology and Transplantation, Abdominal Transplantation, KU Leuven, Leuven, Belgium; Department of Internal Medicine and Paediatrics, Nephrology Section, Ghent University Hospital, Ghent, Belgium; Department of Rehabilitation Sciences, Research Group for Rehabilitation in Internal Disorders, KU Leuven, Leuven, Belgium; Department of Microbiology, Immunology and Transplantation, Nephrology and Renal Transplantation Research Group, KU Leuven, Leuven, Belgium; Department of Cardiovascular Disease, University Hospitals Leuven, Leuven, Belgium; Department of Cardiovascular Sciences, KU Leuven, Leuven, Belgium; Department of Microbiology, Immunology and Transplantation, Nephrology and Renal Transplantation Research Group, KU Leuven, Leuven, Belgium; Department of Nephrology, University Hospitals Leuven, Leuven, Belgium; Department of Public Health and Primary Care, Ghent University, Ghent, Belgium; Department of Internal Medicine and Paediatrics, Nephrology Section, Ghent University Hospital, Ghent, Belgium; Department of Ophthalmology, University Hospitals Leuven, Leuven, Belgium; Department of Neurosciences, Research Group Ophthalmology, KU Leuven, Leuven, Belgium; Biostatistical Centre, KU Leuven, Leuven, Belgium; Department of Internal Medicine and Paediatrics, Nephrology Section, Ghent University Hospital, Ghent, Belgium; Department of Ophthalmology, University Hospitals Leuven, Leuven, Belgium; Department of Neurosciences, Research Group Ophthalmology, KU Leuven, Leuven, Belgium; Department of Microbiology, Immunology and Transplantation, Abdominal Transplantation, KU Leuven, Leuven, Belgium; Department of Microbiology and Immunology, Rega Institute for Medical Research, Leuven, Belgium; VIB-KU Leuven Centre for Microbiology, KU Leuven, Leuven, Belgium; Institute of Nursing Science, Department Public Health, Faculty of Medicine, University of Basel, Basel, Switzerland; Academic Centre for Nursing and Midwifery, Department of Public Health and Primary Care, KU Leuven, Leuven, Belgium; Department of Internal Medicine and Paediatrics, Nephrology Section, Ghent University Hospital, Ghent, Belgium; Department of Microbiology, Immunology and Transplantation, Nephrology and Renal Transplantation Research Group, KU Leuven, Leuven, Belgium; Department of Nephrology, University Hospitals Leuven, Leuven, Belgium; Department of Internal Medicine and Paediatrics, Nephrology Section, Ghent University Hospital, Ghent, Belgium; Department of Rehabilitation sciences, Ghent University, Ghent, Belgium; Department of Microbiology, Immunology and Transplantation, Abdominal Transplantation, KU Leuven, Leuven, Belgium; Transplantoux Foundation, Leuven, Belgium; Department of Rehabilitation Sciences, Research Group for Rehabilitation in Internal Disorders, KU Leuven, Leuven, Belgium; Department of Microbiology, Immunology and Transplantation, Nephrology and Renal Transplantation Research Group, KU Leuven, Leuven, Belgium; Department of Nephrology, University Hospitals Leuven, Leuven, Belgium

**Keywords:** cardiovascular, exercise, kidney transplantation, physical activity, quality of life

## Abstract

**Background:**

Cardiovascular (CV) disease represents a leading cause of death in kidney transplant recipients (KTRs). Poor physical fitness adds to the increased CV risk of KTRs. Exercise-based rehabilitation and physical activity interventions may prove pivotal in both short and long-term outcomes after kidney transplantation.

**Methods:**

PHOENIX-Kidney is a prospective, multicentre, randomized, controlled, single-blinded trial with parallel groups. A total of 147 adult *de novo* KTRs from two independent Belgian transplant centres will be randomized to one of three groups with different exercise intensity: (i) 6 months moderate-intensity aerobic and muscle strengthening exercise training followed by a physical activity intervention (MIT, *n* = 49), (ii) 6 months moderate- and high-intensity aerobic exercise training and moderate-intensity muscle strengthening exercise training followed by a physical activity intervention (MHIT, *n* = 49), or (iii) sham exercise training, not followed by a physical activity intervention (CON, *n* = 49). The training and physical activity interventions are home-based programmes, which will be initiated at 3 and 9 months after transplantation, respectively. Study participants will be followed up until 2 years after transplantation. The primary hypothesis is that peak oxygen uptake (VO_2_peak) assessed after the 6-month home-based training programme (the primary outcome) will increase more in MHIT than in CON. Secondary hypotheses are that VO_2_peak will increase more in MIT compared to CON, and more in MHIT compared to MIT. Secondary endpoints encompass changes in 6-minute walking distance, endothelial function (flow-mediated dilation of the arteria brachialis), health-related quality of life, physical activity, and safety.

**Conclusion:**

PHOENIX-Kidney is the first adequately powered RCT to address the question of optimal training intensity in KTRs. Moreover, the implementation potential of a home-based exercise programme followed by a physical activity intervention will be formally assessed in a real-world clinical setting.

**Clinical Trial Registration:**

Clinicaltrials.gov identifier number: NCT06260579.

KEY LEARNING POINTS **What was known:**

- Both physical inactivity and poor physical fitness, known modifiable cardiovascular risk factors, are highly prevalent after kidney transplantation.
- Although exercise training is safe and beneficial after kidney transplantation, the optimal training intensity remains uncertain.
- Integrating physical activity as part of the daily life after kidney transplantation is highly recommended, but implementation remains a major challenge.

**This study adds:**

- PHOENIX-Kidney will address the question of optimal training intensity after kidney transplantation.
- Once the challenge of establishing the ideal training intensity for KTRs is successfully addressed, it becomes imperative to develop and employ effective implementation strategies to promote sustained physical activity in this patient population. The implementation outcomes in PHOENIX-Kidney provide some answers on this topic.

**Potential impact:**

- The results of this study will contribute to guidelines on optimal training intensity after kidney transplantation. They will also be insightful to develop successful implementation strategies to promote sustained physical activity in this population for improved long-term outcomes.
- The health-economic evaluation has the potential to drive policy changes.

## INTRODUCTION

Kidney transplantation is the treatment of choice for end-stage kidney disease (ESKD) as it improves both quality [1, 2] and quantity [2, 3] of life compared to continued medical care. Advances in the field of transplantation have led to improved post-operative survival rates [4]. However, age-standardized mortality remains 2- to 7-fold higher in kidney transplant recipients (KTRs) [5], with cardiovascular (CV) disease representing a leading cause of death in recipients with a functioning graft [6].

Pre-existing CV disease risk factors such as hypertension, diabetes, left ventricular hypertrophy, anaemia, and mineral bone disease [7], as well as post-transplant compromised graft function [8], lifelong immunosuppressive therapy [9], and physical inactivity [10] condemn KTRs to a 3–5-fold increased risk for CV disease [9]. Modifiable CV risk factors such as physical inactivity, poor physical fitness, low muscle mass, dyslipidaemia, hypertension, obesity, and diabetes are highly prevalent yet inadequately managed in the current medical practice [7, 11]. Twenty to 65% of KTRs eventually develop metabolic syndrome, a well-known risk factor for graft loss and CV mortality in this population [5, 12]. Endothelial dysfunction is the first, yet reversible step towards atherosclerosis [13]. Further progression clinically translates in atherosclerosis and arteriosclerosis (quantified by arterial stiffness measurements) as independent predictors of CV events and mortality in KTRs [14, 15].

Despite increased physical activity levels following kidney transplantation, half of the patients fail to meet physical activity guidelines [16–18] and physical activity levels remain inferior compared to the general population [19]. Accordingly, health-related physical fitness remains largely impaired post-transplant. This is problematic, as both physical activity and impaired physical fitness are established CV risk factors in both the general and transplant population [10, 20–26].

Cardiorespiratory fitness is severely impaired in ESKD and only partially recovers after transplantation to 65%–80% of a matched reference population [11, 27, 28]. Peak oxygen uptake (VO_2_peak) inversely correlates with an adverse CV risk profile and atherosclerotic burden in glucose intolerant KTRs [23]. In fact, VO_2_peak is well-recognized as an independent predictor of all-cause mortality in different populations [29–31], including heart and KTRs [32, 33]. VO_2_peak is a potentially stronger predictor of mortality than smoking, hypertension, hypercholesterolemia, and type 2 diabetes, prompting the American Heart Association to promote routine assessment of cardiorespiratory fitness as a clinical vital sign [34].

Evidence suggests that pathologically decreased muscle mass and function, already present before transplantation in 30%–40% of dialysis patients [35], does not significantly improve within the first 2 years post-transplant [36]. Moreover, persistent or new-onset sarcopenia after transplantation is a common finding [36, 37]. Excessive fat gains lead to a dual disturbance in body composition known as sarcopenic obesity [38]. The excessive fat mass promotes systemic inflammation, whereas the loss in muscle mass contributes to insulin resistance and mitochondrial and endothelial dysfunction. Together they complementary contribute to CV disease development [39].

In conclusion, the pleiotropic effects of exercise training and physical activity are well known in both general and patient populations [39, 40]. Emerging data from smaller randomized clinical trials indicate that exercise training is feasible, safe, and effective in improving physical fitness, some markers of cardiovascular health, and health-related quality (QoL) of life in KTRs [40]. However, the optimal training intensity needed to derive the largest clinical and CV benefits in KTRs is still unknown. In the general population, high-intensity interval training may offer greater adherence and CV benefits compared to moderate-intensity training [41, 42]. Proof of concept data in stable [43] and *de novo* [44] heart transplant recipients show a greater increase in VO_2_peak after high- compared to moderate-intensity training, although without any different response in arterial stiffness or endothelial function [43]. These data warrant the need for a large study investigating the effects of high-intensity interval training in patients with an unfavourable CV risk profile. Once the challenge of establishing the ideal training intensity for KTRs is successfully addressed, it becomes imperative to develop and employ effective implementation strategies to promote sustained physical activity in this patient population, thereby ensuring long-term health benefits.

Through the PHOENIX-Kidney trial, we aim to investigate effectiveness and implementation potential of a 6-month home-based exercise intervention followed by a physical activity behavioural intervention on short and long-term clinical and health-economic outcomes in *de novo* KTRs. We hypothesize that 6 months exercise training will improve VO_2_peak in comparison to a sham control group. The role of exercise intensity will be explored. Finally, the implementation potential of an exercise and physical activity intervention in *de novo* KTRs will be formally assessed in a real-world clinical setting.

## MATERIALS AND METHODS

### Study design and population

**Figure 1:**
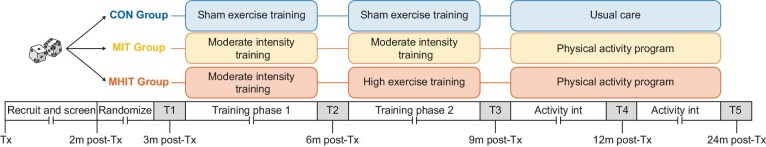
PHOENIX-Kidney study design. CON group: sham exercise training group, MIT group: moderate intensity training group; MHIT group: moderate and high intensity training group; Tx: transplantation; T1: timepoint one; T2: timepoint two; T3: timepoint three; T4: timepoint four; T5: timepoint five.

PHOENIX-Kidney is designed as a prospective, multicentre, randomized, controlled, single-blinded trial with parallel groups (Fig. 1). The research approach has similarities to a hybrid type I effectiveness-implementation research design [45]; next to the evaluation of effectiveness of a home-based exercise and physical activity intervention, information will be gathered on its potential for implementation in real-world clinical settings.

The primary endpoint is VO_2_peak after a 6-month exercise training programme. Secondary endpoints encompass changes in other measurements of physical fitness (6-minute walking distance, 6MWD), endothelial function (flow-mediated dilation of the a. brachialis), health-related quality of life (QoL), physical activity, and safety. Additionally, other measurements of physical fitness and CV health, gut microbiome characteristics, graft function, physical frailty, implementation potential, and cost-effectiveness will be explored at different time points. For the exploratory outcomes, we refer to the supplementary material.

*De novo* KTRs will be recruited from two Belgian tertiary hospitals: University Hospitals Leuven and Ghent University Hospital. Details of the inclusion and exclusion criteria are listed in Table 1. All *de novo* adult KTRs, including re-transplantation, will be screened for eligibility. Written informed consent will be obtained from all participants in the intervention study (ICF-A). A preparticipation medical screening through a maximal cardiopulmonary exercise test (CPET), supervised by a cardiologist, will be performed before randomization.

**Table 1:** Inclusion and exclusion criteria.

Inclusion criteria:
• age >18 years
*• de novo* KTRs
Exclusion criteria:
• underlying heart disease, defined as aberrant CPET, unstable angina, non-revascularized lesions, or life-threatening arrhythmias
• uncontrolled hypertension
• uncontrolled diabetes, defined as HbAlc > 9%
• musculoskeletal disorders not allowing physical training on a cycle ergometer, or any other medical reasons by the physician considered to be a contraindication for moderate or high-intensity training
• multi-organ transplantation
• ongoing malignancies
• unable to understand Dutch
• no access to smartphone and/or computer with internet access
• severe pulmonary disease defined as either FVC < 50%, FEV1 < 50%, or DLCO < 40% that excludes all serious underlying respiratory disease (pulmonary fibrosis, COPD, GOLD II-IV, PAH)

CPET, cardiopulmonary exercise test; FVC, forced vital capacity; FEV1, forced expiratory volume in 1 s; DLCO, diffusing capacity for carbon monoxide; COPD, chronic obstructive pulmonary disease; PAH, pulmonary arterial hypertension; KTR, kidney transplant recipients.

The number and reasons why patients are not eligible or willing to participate will also be recorded. Furthermore, eligible study candidates not willing to participate will be asked whether they would agree to complete questionnaires characterizing their socio-demographic background, medical background, self-reported physical activity, and barriers and motivators to physical activity (ICF-B). These characteristics include age, gender, ethnicity, religion, living situation, informal care situation, family situation, education, work situation, income, smoking behaviour, alcohol consumption, body weight, body height, pre-transplantation dialysis vintage, kidney transplant vintage, type of transplant (cadaveric, living related, living unrelated), cause of kidney failure, estimated glomerular filtration rate (eGFR), and comorbidities. Self-reported physical activity levels will be assessed through the Physical Activity Vital Sign questionnaire [46]. Perceived barriers and motivators to physical activity will be assessed through the Barriers and Motivators Questionnaire [47]. Furthermore, long-term graft survival and mortality will be evaluated also after signing ICF-A.

Ethical approval (B3222022000937) has been obtained from the local ethical committee of UZ Leuven/KU Leuven and U(Z) Ghent. The study complies with the International Conference for Harmonization of Good Clinical Practice guidelines and the declaration of Helsinki.

### Randomization and blinding

Participants will be randomized on an allocation ratio of 1:1:1 to either 6 months of home-based moderate-intensity training followed by a physical activity intervention (MIT; *n* = 49); 6 months of home-based training combining 3 months of moderate and 3 months of high intensity followed by a physical activity intervention (MHIT, *n* = 49); or 6 months of home-based sham exercise training (CON; *n* = 49). A computer-generated randomization sequence, stratified for sex and study centre, will be developed and uploaded to a secure web application (REDCap) used for randomization and data management. Prior to randomization, participants will be clearly informed about the content of each possible intervention arm, including the stipulation that switching groups is not allowed and that adherence to the prescribed training programme is essential throughout the study. Outcome assessors and data investigators will be blinded to the participants’ group allocation, both in University Hospitals Leuven and Ghent University Hospital.

### Study interventions

#### MIT and MHIT groups

All participants in the MIT or MHIT group will take part in a 6-month home-based exercise intervention, followed by a 12-month physical activity intervention.

##### Exercise intervention

The exercise intervention consists of triweekly aerobic training sessions and biweekly training sessions focused on strength, balance, and flexibility.

**Figure 2:**
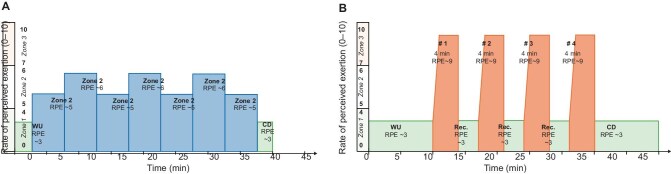
Example of a moderate (**a**) and high (**b**) intensity aerobic training session of equivalent time and training load. RPE: Rate of perceived exertion; WU: warm up; CD: cooling down; Rec: recovery; Zone 1: intensity below the first ventilatory threshold, eliciting RPE of 1-4/10; Zone 2: intensity above the first ventilatory threshold and below the second ventilatory threshold, eliciting RPE of 5-6/10; Zone 3: intensities above the second ventilatory threshold, eliciting RPE of 7-10/10.

For the aerobic training sessions, participants will receive a stationary bicycle at home (Fit bike Ride 5^©^). The MIT group will perform moderate-intensity aerobic training during two training phases of 3 months each. MHIT group will conduct moderate-intensity aerobic training during the first training phase followed by high-intensity aerobic interval training during the second training phase. The moderate-intensity training and high-intensity interval training sessions are of equivalent duration and training load, the latter calculated as the product of duration and rate of perceived exertion (RPE) (Fig. 2).

Aerobic training intensity will be determined using the ‘3-zone training intensity model’ [48], with heart rate training zones extrapolated from the participants’ CPET and corresponding ventilatory thresholds. Zone 1 corresponds to intensities below the first ventilatory threshold, eliciting RPE scores of 1–4/10 on a 0–10 adapted Borg scale [49, 50]. Zone 2 corresponds to intensities above the first ventilatory threshold and below the second ventilatory threshold, resulting in RPE scores of 5–6/10. Zone 3 corresponds to intensities above the second ventilatory threshold, resulting in RPE scores of 7–10/10. Heart rate training zones will be adjusted according to the patient's RPE whenever needed (i.e. RPE scores deviating more than two points from predicted RPE scores on two consecutive training sessions).

**Figure 3:**
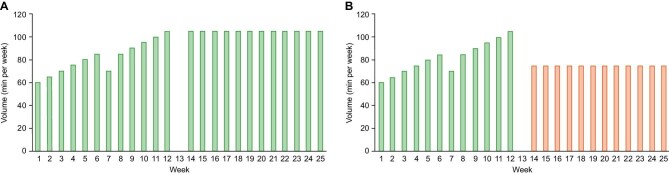
Aerobic training volume in MIT (**a**) group and MHIT (**b**) group. Aerobic training volume in MIT group (A) and MHIT group (B), excluding warming up and cooling down duration. Training volume per high-intensity interval training (25 min) was calculated as 4x4 min high intensity interval + 3x3 min recovery. Green: moderate-intensity training; orange: high-intensity training.

Moderate-intensity training sessions will consist of a 3-min warming up in zone 1, 20–35 min cycling in zone 2, and 2-min cooling down in zone 1 (Fig. 2A). High-intensity interval training will be performed according to the Scandinavian model [44]: 10-min warming up in zone 1, four 4-min bouts in zone 3 resulting in an RPE score of ∼9, each interspersed by 3 min recovery in zone 1 at an RPE score of 3, and a 10-min cooling down in zone 1 (Fig. 2B). The first minute of each of the four 4-min high-intensity exercise bouts will be used to gradually increase intensity from a RPE score of 3 to 9. Weekly aerobic training volume will progressively increase throughout two 6-week mesocycles in training phase 1 and remain constant throughout training phase 2 (Fig. 3).

Strength training will target major muscle groups using elastic bands and body weight exercises (calisthenics) at an intensity inducing muscle fatigue by the end of each set. Each strength training session will consist of a total of 16 exercises, including four warm up exercises with a main focus on active range of motion and flexibility, followed by eight exercises targeting muscle strength and stability, ending with four cooling down stretching exercises.

All participants will receive their personalized training schedules via an available online training platform (Coachbox^©^, Belgium). In this online tool, participants will have access to their personal training account, which displays their training schedule using videos, pictures, and text. Heart rate will be recorded during each training session using a heart rate monitor (Polar Pacer^©^). After the training session, this data will be uploaded to both the Polar Flow platform and Coachbox, either automatically or manually. Moreover, participants will be asked to note their RPE after each aerobic training session and to post comments in their training diary (Coachbox) for them and their coach (a physiotherapist of the research team) to interact. Participants who prefer a paper training diary over the digital training platform will be given a paper diary for completion of RPE scores and comments.

**Figure 4:**
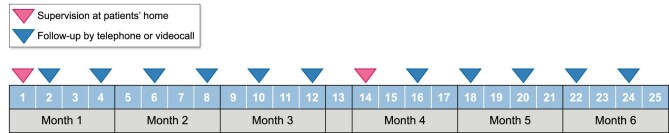
Contact frequency and type throughout the 6-month exercise intervention. All participants receive two initial in-person supervision at home at the begin of each training phase, followed by bi-monthly remote check-ins.

At the beginning of each training phase, in-person supervision will be provided at the participants’ home, followed by twice monthly contacts with the study team via phone call or video conference. However, according to the wishes, needs, and capabilities of the participants, a maximum of 14 in-person supervision at the participants’ home will be provided (Fig. 4).

##### Physical activity intervention

A tailored physical activity intervention will be co-developed using motivational interviewing techniques, taking into account the information of the Motivators and Barriers Questionnaire (see below). A person-centred communication style that respects participants' autonomy and sociocultural context will be used to evoke intrinsic motivation and promote long-term physical activity maintenance [51, 52]. In addition, based on our previous work that identified promising physical activity behaviour change techniques in transplant recipients [53], the following behaviour change techniques (Taxonomy V1 [54]) will be used in the physical activity intervention:

- Antecedents: restructuring physical environment. Participants will be able to keep their stationary bicycle, resistance bands, and heart rate monitor if they want to. Participants will also be able to keep their Coachbox account and embedded training videos and instructions. Other environmental cues (e.g. placing walking shoes in sight) will be customized for each participant.
- Natural consequences. Information about health, emotional, and environmental consequences: Participants and their social contacts (family, friends, health care providers, etc.) will be informed about the benefits and potential adverse outcomes of physical activity and inactivity, respectively. They also will receive information on the preferred mode and frequency of daily physical activity.
- Feedback and monitoring: feedback on behaviour, biofeedback, and self-monitoring of behaviour. Participants will be able to keep their heart rate monitor (smart watch). This device will not only monitor heart rate, but also functions as an accelerometery-based physical activity tracker for self-monitoring of physical activity. Furthermore, the physical activity tracker could provide physical activity cues through visual and/or sensory reminders to be active.
- Goals and planning: behavioural SMART goals setting, action planning, and review of behavioural goals. Participants’ physical activity goals will be personalized to their abilities and preferences. The plan of action will work around physical limitations and patient-reported barriers, while focusing on the participants’ motivators. SMART goals will be established using a stepwise approach that aims for consecutive small successes and habit formation.
- Feedback on behaviour and review of behavioural goals and outcome. Follow-up prompts will be provided during the physical activity intervention.
- Social support: practical, general, and emotional social support. Encouragement and support from the health care team and the participant's social contacts will be promoted.

**Figure 5:**
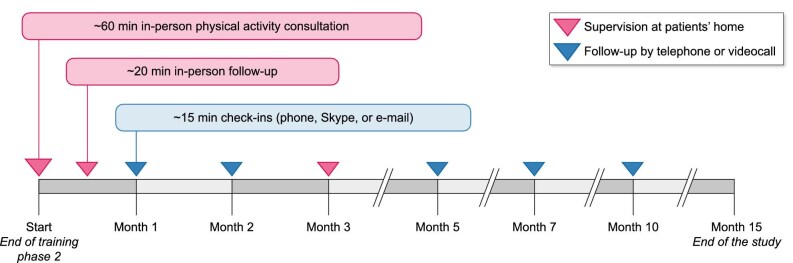
Contact frequency and type throughout the 15-month physical activity intervention. The physical activity intervention is initiated through a 60-minute consultation, 2 in-person follow-ups, and 5 remote prompts, with decreasing frequency over time.

The physical activity intervention will be conceived and evaluated in a face-to-face consultation (60 minutes), two in-person follow-up sessions (20 minutes), and five telephone or videoconference follow-up prompts (15 minutes), delivered at increasing time intervals (Fig. 5).

#### SHAM exercise control (CON)

Participants in the control group will receive a 6-month home-based sham exercise intervention. This intervention will consist of low-intensity balance and flexibility exercise sessions of 20–30 min in duration performed twice a week. No formal physical activity intervention will be delivered after completion of the exercise intervention. Participants will be asked to report information on physiotherapy prescription and physiotherapy attendance if part of their usual care. Training monitoring, using a heart rate monitor (Polar Pacer©, Polar Electro, Finland) with chest strap (H10, Polar Electro, Finland), an online training platform (Coachbox©, Belgium), and supervision provided during the exercise intervention will be similar to the supervision moments in the MIT and MHIT group.

#### Adherence and fidelity

Adherence (the number of performed versus prescribed training sessions) and fidelity (intensity and dose of performed *versus* prescribed exercise) to the exercise intervention will be monitored using a heart rate monitor (Polar Pacer^©^) and an online training diary (Coachbox). This training diary will offer prerecorded videos and pictures, periodized training volume, and personalized training intensities based on participants’ CPET results. If a training cessation of at least 7 days is required, participants will be asked to resume their training regimen at the level it was interrupted before the required training stop.

### Study outcomes

**Figure 6:**
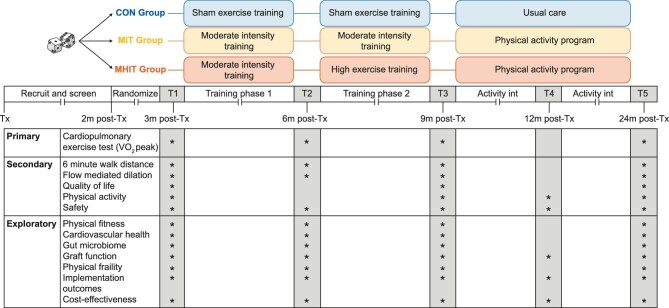
Schematic overview of the participant timeline and study outcomes measured per timepoint.

Outcome parameters will be assessed at five different timepoints: 3 months post-transplant (T1, baseline), 6 months post-transplant (T2), 9 months post-transplant (T3), 1 year post-transplant (T4), and 2 years post-transplant (T5). Figure 6 schematically presents the participant timeline and study outcomes measured per timepoint.

Study procedures will take place at specialized laboratories of both University Hospitals Leuven and Ghent University Hospital, according to prespecified standard operating procedures. For different time points and centres, the order of examinations will be similar, starting with blood sampling, body morphology, and CV tests, all conducted in a fasting state (*cf. infra*). Subsequently, participants will be instructed to take their medication and consume a light breakfast standardized to the participants’ preferences and repeated during follow-up testing. Following this, further examinations will include: motor fitness, cardiorespiratory fitness, musculoskeletal fitness, and the completion of the questionnaires in presence of a member of the research team covering self-reported physical activity, safety, infections, QoL, implementation outcomes, and cost-effectiveness. Immediately after completion of the test day, participants will be requested to start wearing the Actigraph wGT3X-BT accelerometer for seven consecutive days.

All participants will be instructed to maintain an overnight fast (>6 hours) and refrain from caffeine, alcohol, and medication (>12 hours) until the fasting tests have been conducted. Participants will be instructed to refrain from moderate and high-intensity exercise two days prior to each testing day, to minimize acute physical activity-induced interference on the outcome measures.

#### Primary outcome: peak oxygen consumption

A cardiopulmonary exercise test (CPET) will be performed on a cycle ergometer (Ergoline 900, Bitz, Germany) using an incremental ramp protocol [55]. The test will begin with a 3-minute rest period, followed by a series of 10-, 15, or 20-W increments per minute until the participant reaches exhaustion or experiences medical signs or symptoms requiring the test to be stopped. Heart rate will be continuously monitored with a 12-lead ECG, and respiratory data will continuously be measured through breath-by-breath analysis (Oxycon Pro, Jaeger, CareFusion, Germany). Blood pressure will be measured at rest and every 2 minutes during the test. The highest 30-second average of oxygen consumption at the end of the test, defined as VO_2_peak, will be considered the main outcome of the test. Submaximal ventilatory thresholds during a CPET will be determined using a combination of methods [56]. The first ventilatory threshold will be identified by the breakpoint of linearity between minute ventilation and oxygen consumption, the breakpoint of linearity between carbon dioxide production and oxygen consumption (also known as the V-slope or gas exchange threshold), and the first increase in the ratio of minute ventilation to oxygen consumption over time. A second ventilatory threshold will be identified as the second breakpoint of linearity between minute ventilation and oxygen consumption or the first increase in the ratio of minute ventilation to carbon dioxide production over time. Two assessors will independently determine the ventilatory thresholds, and the mean of their values will be taken as the final value.

#### Secondary outcomes

##### Six-minute walking distance

Next to CPET-derived measurements, the six-minute walking test will be performed as a measure of functional cardiorespiratory fitness. During this test, the participant is asked to walk as far as possible within 6 minutes in a 30-m-long corridor, without running, and using walking aids as needed. Participants are motivated and informed about the remaining time in a standardized manner.

##### Endothelial function

Flow-mediated dilation of the brachial artery will be assessed using ultrasound (KU Leuven: UNEX EF, UNEX corporation, Nagoya, Japan; UGhent: ultrasound echo ‘GE Vivid 7’ with the Cardiovascular Suite) as a marker of endothelial function of the large blood vessels. Flow-mediated dilation, defined as the maximal diameter detected within three minutes post-occlusion in relation to the rest diameter (measured before occlusion), will be assessed in a fasted state, supine position, and under standardized conditions, as advised by the expert consensus of Thijssen *et al.* [57]. Offline analyses of the pseudonymized images of the two centres will be performed by one expert, blinded for the randomization arm.

In a subgroup of participants recruited at University Hospitals Leuven, dynamic vessel analysis of the retinal microvasculature (Dynamic Vessel Analyzer, Imedos GmbH, Jena, Germany) will be used to assess endothelial function of the small blood vessels. The details of the assessment procedure have been described elsewhere [58, 59]. Dynamic vessel analysis will not be performed in participants with an anterior chamber depth of <25% of the corneal thickness [60].

##### Quality of life

The Short Form Health Survey (SF-36) is a self-report instrument that will be used to measure limitations in physical, social, and role activities due to poor health and/or bodily pain. It consists of eight categories: physical functioning, role limitations due to physical health, body pain, general health, vitality, social functioning, role limitations due to emotional health, and mental health. These categories are summarized into a physical component score and a mental component score. The ‘Impact on Participation and Autonomy’ questionnaire will be used to measure participants’ ability to participate in activities and make decisions for themselves. It is a valid, reliable, and acceptable measure of participation and autonomy [61]. In-depth semi-structured interviews will be conducted in 29 participants from Ghent University Hospital to explore their experiences with role management and participation during their rehabilitation process. The interviews will be recorded and transcribed. The transcripts will be analysed using Nvivo software to gain insight into the participants’ experiences.

##### Physical activity

Physical activity will be measured using two methods: accelerometery and self-reporting questionnaires. Accelerometery entails wearing an Actigraph wGT3X-BT device at the waist for 7 days to estimate time spent at light, moderate, and vigorous intensity [62]. Participants will be instructed to wear the Actigraph continuously from waking until bedtime for seven consecutive days, immediately starting after completion of the test day. Self-reported physical activity will be measured using the Physical Activity Vital Sign questionnaire, which asks about the days and minutes per week spent in moderate and vigorous physical activity [46].

Perceived barriers and facilitators to physical activity will be measured using the Motivators and Barriers Questionnaire [47]. This questionnaire lists various barriers and facilitators to physical activity and asks the respondent to rate their level of agreement with each item on a four-point scale (from ‘not at all’ to ‘very much’).

##### Safety

An independent Safety and Monitoring Committee will be un-blinded to the randomization groups, monitoring (serious) adverse events during the exercise intervention. Throughout the duration of the study, all participants will be asked to complete a self-developed questionnaire evaluating adverse events and the Immune Status Questionnaire on a monthly basis and at the pre-defined follow-up timepoints to prospectively assess infection incidence and other potential adverse events/reactions [63]. The questionnaire encompasses questions related to infection, hospitalization, musculoskeletal pain, or dysfunction, and hypoglycaemic or cardiorespiratory events. Objectively diagnosed infections for which the study participants sought medical care will be monitored. In addition, long-term graft survival and mortality will be monitored for up to 10 years after inclusion in the study.

### Statistical considerations

#### Sample size calculation

To determine the alpha level, a hierarchy in the hypothesis tests was specified; the comparison of MHIT *vs.* CON will be tested at an alpha level of 0.05. If the previous test was significant, the comparisons of MIT *vs.* CON and MHIT *vs.* MIT will be tested at an alpha level of 0.025. A difference of 2.5 ml·kg^−^^1^·min^−^^1^ in VO_2_peak is considered the minimum clinically important difference [64]. Based on a two-sided analysis of covariance, comparing the mean at the post-intervention time point and correcting for the baseline level of VO_2_peak, a total sample size of 123 is required to have 80% power to detect a difference of 2.5 ml·kg^−^^1^·min^−^^1^ in VO_2_peak between CON and MHIT after the 6-month training intervention (T3). This calculation assumes a standard deviation of 5.0 ml·kg^−^^1^·min^−^^1^ and a 0.7 Rho correlation between the baseline and post-training VO_2_peak levels, based on unpublished data from 20 *de novo* heart transplant recipients from the University Hospitals Leuven. To account for an anticipated dropout rate of 15%, a total of 147 KTRs will be recruited for the present study. If the actual dropout rate exceeds this estimate, additional recruitment will be considered.

#### Statistical analyses

The full analysis set will include all randomized participants and will be used to evaluate all efficacy and safety endpoints according to the intention-to-treat principle. The per protocol set will exclude participants with major protocol deviations and will be reviewed and finalized at a Blind Review Meeting before the database is locked. Only patients with 70% adherence to the prescribed aerobic training sessions will be included in the per protocol analysis. Moreover, before final assessment, patients have to exercise for two consecutive weeks. Participants who do not fulfil these requirements will not be included in the per protocol analysis.

The sample size calculation was based on an analysis of covariance, but a constrained longitudinal data analysis will be used to evaluate the difference in VO_2_peak, taking into account the potential for missing values [65]. The model will be fit to all longitudinal data and will include a fixed effect for centre and sex. Pairwise comparisons between treatment groups will be made using a predetermined hierarchy and will be tested at different alpha levels depending on the comparison being made. The comparison MHIT group *vs.* CON group is first tested at 0.05 and—if this test is significant—the comparisons MIT group *vs.* CON group and MHIT group *vs.* MIT group at 0.025 alpha level.

For continuous secondary outcomes measured longitudinally, the same approach will be used as for the primary outcome, which is a constrained longitudinal data analysis model. For binary secondary outcomes measured longitudinally, a generalized linear mixed model will be applied. The alpha level for testing the significance will be set at 0.05.

## DISCUSSION

Emerging data indicate the feasibility, safety, and effectiveness of exercise training in improving physical fitness, markers of CV health, and QoL in *de novo* KTRs [73]. However, the optimal training intensity for maximizing clinical and CV benefits in KTRs remains unknown. This study will be the first to address this important issue in *de novo* KTRs. Given the low starting training volume and intensity, the gradual increase in training volume over time, and supporting feasibility data from previous studies in the field [40, 44], it is reasonable to expect that the present study intervention will be feasible for most participants.

Home-based programmes with internet-based coaching have been efficient in increasing physical activity and improving fitness in both cardiology and nephrology populations [66, 67]. Pilot studies show that home-based moderate- and high-intensity training is safe and improves VO_2_peak in stable transplant recipients [68]. Importantly, remotely mediated and guided home-based exercise training offers advantages over traditional ambulatory rehabilitation by reducing contamination risk, resource requirements, financial costs, and enabling support for patients regardless of proximity to the transplant centre [69–72]. However, user-friendly tracking of physical activity, regular feedback from exercise professionals, and initial in-person support may be necessary to address barriers such as insecurity, lack of confidence, and motivation [73]. Against this background, the PHOENIX-Kidney study was developed as a hybrid form of home-based training with gradually decreasing in-person supervision and gradually increasing telerehabilitation (remotely delivered consultations), which most likely represents the optimal delivery mode for post-kidney transplantation physical rehabilitation interventions. By combining home-based training with digital monitoring and professional support, the study aims to bridge the gap between hospital rehabilitation and long-term physical activity. Evaluating implementation and cost-effectiveness will provide essential insights into its feasibility and inform future healthcare policies. In conclusion, it is clear that a personalized approach is necessary to successfully complete exercise interventions in complex populations such as KTRs but also to develop physical activity programmes for a sustainable effect. In PHOENIX-Kidney, the efficacy of a home-based exercise programme and physical activity intervention will be evaluated for improving physical fitness, endothelial function, QoL, physical activity, and safety. This two-phase RCT will provide new insights into the role of exercise intensity in enhancing cardiorespiratory fitness, a key clinical variable in KTRs that inversely correlates with CV risk and atherosclerotic burden [23]. By improving cardiorespiratory fitness, this intervention may help reduce CV disease risk, the leading cause of death in KTRs [6]. As the first adequately powered multicentre RCT assessing basic, clinical, and health-economic outcomes in this context, PHOENIX-Kidney could serve as a blueprint for integrating exercise into routine post-transplant care, ultimately improving long-term health outcomes.

The PHOENIX-Kidney study has some limitations. First, participation requires a certain level of digital and technical literacy, as patients must use a smartphone and a smart watch (Polar Pacer^©^). While we provide in-person support, video-based instruction, and manuals, this requirement may still limit generalizability. Second, no formal usual care group is included. The CON group receives a sham exercise training consisting of low-intensity training. This approach was chosen because usual care for KTRs in Belgium is evolving, leading to significant variability across hospitals and nephrologists. To account for these differences and ensure a more structured comparison, a standardized low-intensity training programme was included as the sham intervention. Last, the strict inclusion and exclusion criteria, including the restriction to Dutch-speaking patients, may affect the study's reach.

## Supplementary Material

sfaf114_Supplemental_File

## Data Availability

No new data were generated or analysed in support of this research.
