# Supplementary material for: Home-based exercise and physical activity intervention after kidney transplantation: impact of exercise intensity (PHOENIX-Kidney). Protocol for a multicentre randomized controlled trial
Source: Clin Kidney J. 2025 Apr 24;18(11):sfaf114. doi: 10.1093/ckj/sfaf114 (PMC12596492; doi:10.1093/ckj/sfaf114)
Supplement: sfaf114_Supplemental_File [file sfaf114_supplemental_file.docx]

**Supplement on**

**Home-based exercise and physical activity intervention after kidney transplantation:
impact of exercise intensity [PHOENIX-Kidney]**

***protocol for a multicentre randomized controlled trial***

##### *Exploratory study outcomes*

##### **Physical fitness**

##### Musculoskeletal fitness

Isometric **handgrip strength** of the dominant hand (to be as standardized as possible [1]) will be measured using a Jamar Hydraulic Hand Dynamometer (Sammons Preston Inc.). For participants with an arteriovenous fistula, the side of the fistula (usually the non-dominant side) will be documented. The measurement will be conducted in a seated position with the elbow flexed at 90°. Measurements will be taken in triplicate and the best result out of three attempts will be used. **Lower muscle strength** will be assessed by measurement of knee extensor muscle strength of the dominant leg via an isometric dynamometer (Biodex Medical Systems Inc., 840-000 System 4, New York, USA) [2]. The study participants will perform three 6-second maximal voluntary isometric contractions at a 60-degree angle of the knee, with 60 seconds of passive recovery between attempts. The highest value, also known as peak torque, will be recorded as the maximal isometric strength. Knee extensor **muscle endurance** of the dominant leg will be assessed using an isokinetic dynamometer (Biodex Medical Systems Inc., 840-000 System 4, New York, USA). The study participant is asked to perform two sets of 25 consecutive maximal isokinetic knee extensions at a speed of 180 degrees per second, with 120 seconds of recovery between sets. The mean torque and percentage decrement score of the best set will be used as marker for muscle endurance [3].

##### Motor fitness

The Short Physical Performance Battery (SPPB), a widely used tool for assessing functional performance or status, will be used to measure motor fitness. It consists of 3 components: postural balance, walking speed, and chair stand tests. Each component is scored on a scale of 0 to 4, for a total composite score of 0 to 12. A higher score indicates better functional status, and a score of less than 10 indicates impairment [4, 5].

##### Body morphology

Anthropometric characteristics such as body weight, body height, and waist and hip circumference will be measured in a fasted state using standard equipment. In addition, in participants from Leuven, body composition will be measured in a fasted state in supine position using Bodystat 1,500 (Bodystat Ltd, Douglas, Isle of Man, UK) [6]. In participants from Ghent, body composition will be measured by means of a DXA scan (Discovery W – Hologic Inc., Bedford, MA). More specific, the four-compartment (4C) model will be used, which gives a valid and reliable estimation of percentage body fat, fat mass, and lean mass [7].

1. **Cardiovascular health**

##### Blood profile

Venous blood samples will be taken after an overnight fast and will be used to assess CV risk profile. This will include glucose homeostasis (measured through fasting blood glucose, fasting blood insulin, Homeostatic Model Assessment for Insulin Resistance (HOMA-IR), and HbA1c), blood lipid profile (triglycerides and cholesterol), and systemic inflammation (measured through high-sensitivity C-reactive protein).

##### Blood pressure

In-office blood pressure will be measured in a fasted state and in triplicate using an automatic device (Omron M6, Omron Health-care Europe, Milton Keynes, UK).

##### Arterial stiffness

Carotid-femoral pulse wave velocity (cf-PWV) will be used as a marker of arterial stiffness (KU Leuven: SphygmoCor device, XCEL model, AtCor Medical, Australia; UGhent: SphygmoCor device, CVMS CVP model, AtCor Medical, Australia). Sphygmocor CVMS device uses applanation tonometry, sequentially at the carotid and femoral artery gated by a simultaneously recorded ECG signal [8]. The more recent SphygmoCor XCEL device eliminates the use of ECG signal by means of leg cuff detection of the femoral pulse in conjunction with carotid tonometry, measuring both proximal and distal sites simultaneously [8]. All measurements will be performed in a supine position, at the contralateral site of the transplanted kidney, in a fasted state, and in triplicate, and will be repeated when they do not meet the quality control guidelines as defined by the manufacturer.

1. **Gut microbiome characteristics**

On test days T1, T2, T3, and T5, all participants will be provided with a stool sampling kit. Following each test day, participants will be instructed to collect a stool sample at home within the subsequent week and complete the accompanying gut microbiome co-variates questionnaire.

The assessment of gut microbiome composition and diversity will involve quantitative and relative microbiome profiling through Fecal 16S rRNA sequencing, supplemented by shotgun metagenomics where possible. This analysis encompasses phylogenetic composition at various taxonomic levels, construction of orthology-based functional profiles, and characterization of microbial metabolic potential. Enterotypes will be identified via Dirichlet Multinomial Mixtures, cell counts will be obtained through flow cytometry, and fecal samples will be processed for dry weight determination. Gut inflammatory status will be assessed through fecal calprotectin levels, and short-chain fatty acid concentrations will be analyzed via gas chromatography. The data will be analyzed using these state-of-the-art approaches, which have been applied in previous research projects [9–14].

In addition, covariates will be considered in the analysis of the gut microbiome data through distance-based redundancy analyses [10–14]. These analyses will use participant metadata (such as information collected through a questionnaire or medical records) on the participant's medical history, lifestyle (amongst others physical activity and diet), well-being, and other events that could affect the composition and activity of the gut microbiome.

Statistical analyses of the gut microbiome outcomes will be conducted using the open-source statistical platform R and the packages vegan, phyloseq, and coda.base. Samples will be clustered into community types using the Dirichlet multinomial mixtures method [15]. The sequencing data will be combined with flow cytometric assessment of microbial loads to obtain quantitative microbiome profiles [10], which provide more information than just composition and are not constrained by compositional statistical methods. Statistical analyses will include the estimation and comparison of microbiome diversity indices, overall composition comparisons using ordination methods, between-group analyses, and correlations with host metadata while considering known confounders such as weight loss and metabolic health. Multiple testing correction using the Benjamini-Hochberg FDR method will be performed when applicable.

1. **Graft function**

Markers of kidney function will include creatinine-based estimated glomerular filtration rate (eGFR), cystatin C-based eGFR, 24-hour urine creatinine clearance, and proteinuria. In a subgroup of participants of University Hospitals Leuven, (sub-)clinical kidney transplant rejection will be assessed in protocol biopsies at 3, 12 and 24 months after transplantation. However, protocol biopsies are part of routine standard of care and will be performed in the same week of T1, T4 and T5 but not at the same day.

1. **Physical frailty**

The Fried's Frailty Phenotype (FFP) score is a validated method for assessing frailty that considers five components: weight loss, exhaustion, physical activity, grip strength, and walking speed. The first two components, weight loss and exhaustion, will be questioned by the assessor. However, the last three components, physical activity, grip strength and walking speed, are part of other outcome parameters, i.e., Physical Activity Vital Sign questionnaire, hand grip strength dynamometry, and Short Physical Performance Battery test. Participants with one or two of these factors are considered pre-frail, and those with three or more are considered frail [16].

1. **Implementation potential**

Implementation outcomes that will be evaluated in the present study: Safety: Number and type of adverse events/reactions and serious adverse events/reactions reported using self-developed questionnaires and/or occurring during the study period. Eligibility: Number of eligible KTRs versus total number of patients receiving a kidney transplantation at the University Hospitals Leuven and Ghent University Hospital throughout this study's recruitment period. Also, the reasons for not being eligible will be monitored; Recruitment: Number of eligible study candidates willing to participate *versus* total number of eligible KTRs, assessed throughout the entire study recruitment period; Attrition: The number of participants who dropout from the study will be monitored. Exit interviews at the time of study cessation will be conducted to describe participants’ experience of study participation and reasons for dropout; Training adherence: The number of performed *versus* prescribed exercise sessions, assessed by registration of training heart rate data in an online training diary (Coachbox). Cut-offs for good, acceptable, and poor adherence are arbitrarily set at ≥80%, 79 to 60%, and <60%, respectively; Training fidelity: Intensity and dose of performed *versus* prescribed aerobic exercise, assessed by analysis of training heart rate; Physical activity initiation: Number of participants co-developing (9 months posttransplant) and initiating a personalized physical activity program (12 months posttransplant); Physical activity maintenance: Number of participants adhering to the physical activity intervention, objectively assessed using accelerometry at 24 months posttransplant; Acceptability, appropriateness, and feasibility: Measured by recruitment, attrition, adherence, fidelity, and one-on-one in-depth semi-structured interviews conducted at the moment of study dropout or completion.

1. **Cost-effectiveness**

The iMTA medical consumption questionnaire (iMCQ) and the iMTA productivity cost questionnaire (iPCQ) will be used to collect data on the number of visits to a general practitioner, specialist, or hospital, the number of sick leave days, and the number of days of reduced productivity due to health problems [17]. This information will be used to calculate the cost of medical consumption and productivity loss. The EQ-5D-5L, a concise questionnaire evaluating health-related QoL, is one of the most commonly used and preferred instruments for health economic evaluations [18]. It consists of five dimensions: mobility, self-care, usual activities, pain/discomfort, and anxiety/depression. Each dimension will be scored on a 3-point Likert scale [19, 20]. The EQ-5D will be used to calculate incremental cost-effectiveness ratios, which compare the costs and benefits of different interventions. More specific, the incremental cost-effectiveness ratio will be calculated by dividing the difference in costs between the study arms by the difference in quality-adjusted life years (QALY) between the study groups. The cost-effectiveness acceptability curve will show the probability that the intervention is cost-effective at different willingness-to-pay thresholds.

**References**

[1] Delanaye P, Quinonez K, Buckinx F, et al. Hand grip strength measurement in haemodialysis patients: Before or after the session? *Clin Kidney J* 2018; 11: 555–558.

[2] Kim WK, Kim DK, Seo KM, et al. Reliability and validity of isometric knee extensor strength test with hand-held dynamometer depending on its fixation: A pilot study. *Ann Rehabil Med* 2014; 38: 84–93.

[3] Girard O, Mendez-Villanueva A, Bishop D. Repeated-sprint ability part I: Factors contributing to fatigue. *Sport Med* 2011; 41: 673–694.

[4] de Villar LOP, Martínez-Olmos FJ, Junqué-Jiménez A, et al. Test-retest reliability and minimal detectable change scores for the short physical performance battery, one-legged standing test and timed up and go test in patients undergoing hemodialysis. *PLoS One*; 13. Epub ahead of print 1 August 2018. DOI: 10.1371/journal.pone.0201035.

[5] Basu A. Role of Physical Performance Assessments and Need for a Standardized Protocol for Selection of Older Kidney Transplant Candidates. *Kidney Int Reports* 2019; 4: 1666–1676.

[6] Buchholz AC, Bartok C, Schoeller DA. The validity of bioelectrical impedance models in clinical populations. *Nutr Clin Pract* 2004; 19: 433–446.

[7] Smith-Ryan AE, Mock MG, Ryan ED, et al. Validity and reliability of a 4-compartment body composition model using dual energy x-ray absorptiometry-derived body volume. *Clin Nutr* 2017; 36: 825–830.

[8] Stabouli S, Printza N, Zervas C, et al. Comparison of the SphygmoCor XCEL device with applanation tonometry for pulse wave velocity and central blood pressure assessment in youth. *J Hypertens* 2019; 37: 30–36.

[9] Vandeputte D, Falony G, Vieira-Silva S, et al. Stool consistency is strongly associated with gut microbiota richness and composition, enterotypes and bacterial growth rates. *Gut* 2016; 65: 57–62.

[10] Vandeputte D, Kathagen G, D’Hoe K, et al. Quantitative microbiome profiling links gut community variation to microbial load. *Nature* 2017; 551: 507–511.

[11] Vieira-Silva S, Sabino J, Valles-Colomer M, et al. Quantitative microbiome profiling disentangles inflammation- and bile duct obstruction-associated microbiota alterations across PSC/IBD diagnoses. *Nat Microbiol* 2019; 4: 1826–1831.

[12] Vieira-Silva S, Falony G, Belda E, et al. Statin therapy is associated with lower prevalence of gut microbiota dysbiosis. *Nature* 2020; 581: 310–315.

[13] Bryan S, Davis J, Broesch J, et al. Choosing your partner for the PROM: A review of evidence on patient-reported outcome measures for use in primary and community care. *Healthc Policy* 2014; 10: 38–51.

[14] Falony AG, Joossens M, Wang J, et al. Title : Population - level analysis of gut microbiome variation.

[15] Holmes I, Harris K, Quince C. Dirichlet multinomial mixtures: Generative models for microbial metagenomics. *PLoS One*; 7. Epub ahead of print 3 February 2012. DOI: 10.1371/journal.pone.0030126.

[16] Fried LP, Tangen CM, Walston J, et al. Frailty in older adults: Evidence for a phenotype. *Journals Gerontol - Ser A Biol Sci Med Sci* 2001; 56: 146–157.

[17] Bouwmans C, Krol M, Severens H, et al. The iMTA Productivity Cost Questionnaire: A Standardized Instrument for Measuring and Valuing Health-Related Productivity Losses. *Value Heal* 2015; 18: 753–758.

[18] Kennedy‑Martin M, Slaap B, Herdman M, et al. Which multi‑attribute utility instruments are recommended for use in cost‑utility analysis? A review of national health technology assessment (HTA) guidelines. 2020; 1245–1257.

[19] Van Wilder L, Charafeddine R, Beutels P, et al. Belgian population norms for the EQ-5D-5L, 2018. *Qual Life Res* 2021; 31: 527–537.

[20] Cleemput I, Kesteloot K, Moons P, et al. The construct and concurrent validity of the EQ-5D in a renal transplant population. *Value Heal* 2004; 7: 499–509.
